# Differential Biases and Variabilities of Deep Learning–Based Artificial Intelligence and Human Experts in Clinical Diagnosis: Retrospective Cohort and Survey Study

**DOI:** 10.2196/33049

**Published:** 2021-12-08

**Authors:** Dongchul Cha, Chongwon Pae, Se A Lee, Gina Na, Young Kyun Hur, Ho Young Lee, A Ra Cho, Young Joon Cho, Sang Gil Han, Sung Huhn Kim, Jae Young Choi, Hae-Jeong Park

**Affiliations:** 1 Department of Otorhinolaryngology Yonsei University College of Medicine Seoul Republic of Korea; 2 Center for Systems and Translational Brain Sciences Institute of Human Complexity and Systems Science Yonsei University College of Medicine Seoul Republic of Korea; 3 Graduate School of Medical Science, Brain Korea 21 Project, Department of Nuclear Medicine Yonsei University College of Medicine Seoul Republic of Korea; 4 Department of Emergency Medicine Yonsei University College of Medicine Seoul Republic of Korea

**Keywords:** human-machine cooperation, convolutional neural network, deep learning, class imbalance problem, otoscopy, eardrum, artificial intelligence, otology, computer-aided diagnosis

## Abstract

**Background:**

Deep learning (DL)–based artificial intelligence may have different diagnostic characteristics than human experts in medical diagnosis. As a data-driven knowledge system, heterogeneous population incidence in the clinical world is considered to cause more bias to DL than clinicians. Conversely, by experiencing limited numbers of cases, human experts may exhibit large interindividual variability. Thus, understanding how the 2 groups classify given data differently is an essential step for the cooperative usage of DL in clinical application.

**Objective:**

This study aimed to evaluate and compare the differential effects of clinical experience in otoendoscopic image diagnosis in both computers and physicians exemplified by the class imbalance problem and guide clinicians when utilizing decision support systems.

**Methods:**

We used digital otoendoscopic images of patients who visited the outpatient clinic in the Department of Otorhinolaryngology at Severance Hospital, Seoul, South Korea, from January 2013 to June 2019, for a total of 22,707 otoendoscopic images. We excluded similar images, and 7500 otoendoscopic images were selected for labeling. We built a DL-based image classification model to classify the given image into 6 disease categories. Two test sets of 300 images were populated: balanced and imbalanced test sets. We included 14 clinicians (otolaryngologists and nonotolaryngology specialists including general practitioners) and 13 DL-based models. We used accuracy (overall and per-class) and kappa statistics to compare the results of individual physicians and the ML models.

**Results:**

Our ML models had consistently high accuracies (balanced test set: mean 77.14%, SD 1.83%; imbalanced test set: mean 82.03%, SD 3.06%), equivalent to those of otolaryngologists (balanced: mean 71.17%, SD 3.37%; imbalanced: mean 72.84%, SD 6.41%) and far better than those of nonotolaryngologists (balanced: mean 45.63%, SD 7.89%; imbalanced: mean 44.08%, SD 15.83%). However, ML models suffered from class imbalance problems (balanced test set: mean 77.14%, SD 1.83%; imbalanced test set: mean 82.03%, SD 3.06%). This was mitigated by data augmentation, particularly for low incidence classes, but rare disease classes still had low per-class accuracies. Human physicians, despite being less affected by prevalence, showed high interphysician variability (ML models: kappa=0.83, SD 0.02; otolaryngologists: kappa=0.60, SD 0.07).

**Conclusions:**

Even though ML models deliver excellent performance in classifying ear disease, physicians and ML models have their own strengths. ML models have consistent and high accuracy while considering only the given image and show bias toward prevalence, whereas human physicians have varying performance but do not show bias toward prevalence and may also consider extra information that is not images. To deliver the best patient care in the shortage of otolaryngologists, our ML model can serve a cooperative role for clinicians with diverse expertise, as long as it is kept in mind that models consider only images and could be biased toward prevalent diseases even after data augmentation.

## Introduction

Machine learning (ML) based on deep learning (DL) in medical imaging is developing at a rapid pace, to fill the gap between the capacity of specialists interpreting the images and the need for interpreted images. Many studies [1-6] show the possibility that the performance of image classification is on par or better than that of medical specialists in terms of accuracy. Despite the promising results of these studies, characteristics of DL have not been thoroughly evaluated and compared with human experts, particularly in the domain of clinical practice. In tasks such as medical image diagnosis, where accountability is an important issue, cooperation between human experts and ML models is necessary [1]. To foster cooperation between humans and machines, the characteristics of human intelligence (HI) and DL-based artificial intelligence (AI) should be specified at the individual and systemic levels.

The class imbalance in real-world clinics is a big challenge in data-driven ML. Different numbers of samples in various classes due to imbalanced incidences inherent in the human population are expected to induce biases toward high incident classes during the training process.

Conversely, human medical experts learn in-depth by experiencing limited numbers of cases, thus have less bias for classes of different sizes [7]. However, clinical experience differs among clinicians, and every clinician has their own classification biases, that is, strengths and weaknesses in classifying certain diseases [8]. Due to the bias induced by individual experience, physicians may have large interindividual variability. Meanwhile, ML models are statistically biased based on the amount of data but show consistent performance among different models [9]. Despite general speculations, these 2 biases for data size for each class and interindividual variation due to differential (small sample–biased) experiences have not been directly evaluated in the clinical diagnostic setting.

In this study, we investigated the differential characteristics of ML models and human experts concerning class imbalance bias and interrater variability. For this, we use as the example the classification of ear and mastoid disease using otoendoscopic images. Ear and mastoid diseases are common in, but not limited to, developing countries in Southeast Asia, Western Pacific regions, and Africa [10]. However, otolaryngologists are shorthanded in many developing countries, with as few as <1 otolaryngologist per a million people in 64% of African counties [11]. Therefore, nonotolaryngologists in primary care are likely to see patients with these diseases in clinics, and they must play a role in managing ear diseases, particularly in areas with limited access to otolaryngologists. However, nonotolaryngologists are prone to misdiagnosing otitis media, which is a major part of ear disease [11-13]. Evaluating ear disease involves careful history taking and physical examination using conventional otoscopy or otoendoscopy. The initial impression of otoscopy is an essential gateway to diagnosis and treatment.

One of the domain-specific challenges in ear disease classification, as in other medical fields, is the class imbalance problem discussed earlier. This problem may affect both clinicians and ML models but possibly more so ML models. Because ear diagnosis is conducted by clinicians with diverse levels of expertise, the variability of individual performance is apparent in this field [14,15].

To investigate and compare the effect of the class imbalance problem between human physicians and ML models as well as interindividual variability, we evaluated the diagnostic rate and interrater reliability of otoendoscopic images among 3 groups: otolaryngologists (2 specialists and 4 residents), nonotolaryngologists (2 family medicine specialists, 2 emergency medicine specialists, and 5 general practitioners), and 13 convolutional neural network (CNN)–based classification models in both balanced and imbalanced test sets, each containing 300 otoendoscopic images. We also examined the dependency of the accuracy on the prevalence of each class in the machines compared with that of human experts. The class imbalance problem was evaluated concerning diverse data augmentation strategies generalizable for most CNN-based classification models to overcome the aforementioned class imbalance problem. We also evaluated the effect of the augmentation strategy in improving classification accuracy according to the incidence of the disease. All these evaluations were conducted by optimizing our previous automated diagnosis system [9]. Furthermore, we sought the possibility of using our classification system as a virtual otolaryngologist to assist physicians by comparing the accuracy and likelihood of diagnosis between our classification system and otolaryngologists.

## Methods

### Patient Data Selection and Acquisition

Digital otoendoscopic images from patients who visited the outpatient clinic in the Department of Otorhinolaryngology at Severance Hospital, Seoul, South Korea from January 2013 to June 2019 were used. A total of 22,707 otoendoscopic images routinely taken using different otoendoscopic cameras by otolaryngology residents, faculty, or experienced nurses were reviewed for labeling. The image resolution was 640 x 480 pixels in the DICOM format. We excluded postsurgerical status photos, duplicate images, images that were significantly out of focus or fuzzy, and otoendoscopic images from the same patient’s follow-up data without changes in the diagnosis. We aggressively excluded similar images if an image was taken multiple times at slightly different angles; we selected only one of the images. As a result, 7500 otoendoscopic images were selected for labeling. This study was approved by the Severance Hospital Institutional Review Boards (IRB No 2019-0467-001). Written informed consent was obtained from physician participants. All methods complyed with the Declaration of Helsinki.

### Analysis and Labeling of Otoendoscopic Images

Otoendoscopic photos containing eardrums and the external auditory canal (EAC) were classified into 6 categories to cover all diseases based on the *Color Atlas of Endo-Otoscopy* [16]: (1) normal eardrum and EAC including healed perforation and tympanosclerosis; (2) tumorous condition, in which there are tumors in the middle ear, EAC, or cerumen impaction; (3) otitis media with effusion; (4) myringitis or otitis externa; (5) perforated eardrums; (6) attic retraction or middle ear atelectasis. Internally, there were more subclasses, but we consequently merged those subclasses into the 6 aforementioned classes because we could not acquire an adequate number of sample sizes of smaller subclasses. Since the goal of the diagnosis system is to offer an appropriate treatment strategy in real-world clinics, the label was constructed considering both required treatment and the similarity of physical findings.

Often, there could be multiple etiologies present in 1 otoendoscopic image. For example, attic retraction with middle ear effusion could be present. In such cases, the image was labeled as attic retraction according to our labeling priority. This priority was determined by the certainty of disease and possible need for surgery.

To ensure the ground-truth label was correct, we applied additional steps in labeling, since the accuracy of otoscopy by a single physician may only be 75% [17]. First, all images were double-checked by reviewing the patient’s diagnosis in the electronic medical record by the attending physician at the time, who had at least 10 years of clinical experience in a tertiary referral center. Second, if the otoendoscopic image was not trivial, even after reviewing the medical records, additional test results (audiological tests including pure-tone audiometry and impedance audiometry, radiological tests including computed tomography, magnetic resonance imaging) were considered for labeling the ground truth. Last, if the first author could not agree or make an appropriate impression on the otoendoscopic image even after combining medical records and additional tests, the picture was discarded. An in-house graphic user interface software built with MATLAB2019a (MathWorks Inc, Natick, MA) was used for manual labeling.

### Supervised Training of CNN Models for EAC Data With Transfer Learning

Public CNN models were pretrained with the ImageNet database [18] to classify 1000 natural objects that served as a base model for transfer learning of otoendoscopic images. Pareto-efficient models were chosen to be transferred to this study domain. They were ResNets [19] (ResNet101, ResNet152), InceptionV3 [20], InceptionV4 [21], Inception-ResNet-V2 [21], VGG-19 with batch normalization [22], SENet [23], DenseNet [24], and NASNet [25,26]. Those models were modified to classify 6 categories of otoendoscopic images by replacing the last fully connected layer of each model with a layer of 6 fully connected output nodes. For model optimization, Adaptive Moment Estimation (ADAM) [27] with a batch size of 32 was used. Larger batch sizes were not used according to a study reporting the advantage of smaller batch sizes [28]. We trained for a total of 20 epochs with differential learning rates. The initial learning rate was 0.01 in the last transferred layer for 5 epochs. After 5 epochs, fine-tuning was done: All the layers were trained for 7 epochs with a discriminative learning rate, ranging from 1x10^-4^ in the last layer to 1x10^-6^ in the first layer. Afterward, we trained for 7 epochs with a learning rate of 1x10^-9^ in the last layer and 3.3x10^-10^ in other layers. To prevent overfitting, affine transformations of images were applied. A horizontal flip, rotation of up to 20 degrees, random scales between 0.8 and 1.2, change of lighting up to 20%, and a random symmetric warp of magnitude between –0.2 and 0.2 were randomly applied with a probability of 75% on every epoch. Model construction, training, validation, and testing were implemented using Pytorch [29] with the Fastai library [30].

### Comparison of the Accuracy of the Models With Diverse Training Settings

#### Comparison of Model Construction and Performance According to Training Sample Size

Among a total of 7500 otoendoscopic images, 7200 images (300 mutually exclusive images were left out for testing in both balanced and imbalanced scenarios; Table 1) were used for training in 20 epochs. To maximize available data for training, we included data from other test sets into the training set; that is, we put the imbalanced test dataset into the training set when evaluating in the balanced testing environment and vice versa when evaluating in the imbalanced testing environment.

**Table 1:** Composition of the training and test sets as well as labels, sorted by labeling priority.

Classification	Number of images		
	Training (n=6900), n (%)	Test-balanced^a^ (n=300), n (%)	Test-imbalanced^b^ (n=300), n (%)
(1) Tympanic perforation	1793 (26.99)	50 (16.77)	51 (17.00)
(2) Attic retraction/atelectasis	521 (7.56)	50 (16.77)	20 (6.67)
(3) Myringitis/otitis externa	256 (3.71)	50 (16.77)	15 (5.00)
(4) Otitis media with effusion	506 (7.33)	50 (16.77)	29 (9.67)
(5) Tumors	285 (4.13)	50 (16.77)	18 (6.00)
(6) Normal	3539 (51.29)	50 (16.77)	167 (55.67)

^a^All classes are distributed equally.

^b^Classes are distributed proportionally to the training set.

We chose random image samples using different random seeds 5 times to flatten accuracy fluctuations. Performance according to training sample size was evaluated to verify the significance of the larger training sample size: 10% (720 images), 25% (1800 images), 50% (3600 images), 90% (6480 images), and 100% (7200 images).

#### Strategies to Overcome Class Imbalance Between Labels

Class imbalance was inevitable due to the diverse incidence of various ear diseases. To mitigate this problem, 3 strategies were incorporated in training: oversampling, the mixup [31] method, and focal loss [32] as the loss function (focal loss with γ = 1). Oversampling was done by copying images in the smaller classes to a level equivalent to the largest class, combined with affine transformations of images. Images of diseases other than normal eardrums were oversampled to reach the number of normal eardrum images in the current database. Images of otitis media with effusion and attic retractions were augmented approximately 6-fold. The images of myringitis and tumors required almost 10-fold oversampling. Mixup and focal loss are described in detail in Multimedia Appendix 1.

We tested 12 models with 8 different configurations (baseline, with and without oversampling, focal loss, and mixup) resulting in a total of 12 x 2 x 2 x 2 = 96 CNN-based ML model variants.

#### Evaluation of the ML Model Accuracy and Similarities in Prediction Tendency in Both Balanced and Imbalanced Test Sets

After fine-tuning various CNN-based ML models, the accuracies of all models were evaluated in both balanced and imbalanced testing scenarios (Table 1). The first, balanced, 300-image set consisted of 50 images for each label, which is different from the incidence ratio in clinical settings but better suited for measuring accuracies. The second, imbalanced, 300-image set contained different numbers of images with each label based on its prevalence in the database, which may represent the proportion of disease in real-world clinics, particularly a tertiary referral hospital. Also, the likelihood of diagnosis between different ML models was evaluated using the Fleiss kappa method [33]. The kappa (κ) scores were interpreted as follows: κ<0 as poor, 0.01-0.20 as slight, 0.21-0.40 as fair, 0.41-0.60 as moderate, 0.61-0.80 as substantial, and 0.81-1 as almost perfect agreement [34].

### Evaluation of Human Physicians’ Diagnoses: Accuracy and Variability

A computerized online questionnaire consisting of 2 sets of 300 questions, identical to the ML model’s balanced and imbalanced test sets (600 images in total, Table 1), was presented to 14 participants in 3 groups: 6 otolaryngologists (2 otolaryngologists, 4 otolaryngology residents), 8 nonotolaryngologists who had previous exposure to otoscopy (2 emergency medicine specialists, 2 family medicine specialists), and 4 general practitioners). Informed written consent was obtained from all participants.

All participants answered the questionnaire in the same order. Participants were requested to answer according to the same labeling priority as in ML models if more than one pathology was present in the given image. Along with the diagnosis, the participants were asked to rate the confidence of their diagnosis on a scale of 1 (not confident) to 10 (very confident). The participants were not told whether the set was balanced or imbalanced, since it might have provided additional clinical suspicion of less common disease entities.

Interrater agreement among individual groups was calculated using the aforementioned Fleiss kappa method [33]. Spearman correlation analyses were also performed to check the possible relationships between confidence and accuracy of diagnosis to determine whether higher confidence is associated with better accuracy.

### Comparison of Diagnostic Performance and Tendency Between Physicians and ML Models

All the answers, which were provided in identical order, from the human physicians and ML models were lined up to compare the accuracy. We evaluated the differences in the classification pattern depending on the class prevalence between physicians and ML models in both balanced and imbalanced test sets. We measured the likelihood of the ML model’s diagnosis to that of human physicians by comparing kappa values. We also compared the per-class accuracy, precision, recall, and F1 scores between physicians and ML models. We then analyzed the differential effects of class prevalence in accuracy and prediction counts using linear regression analysis.

## Results

### Training and Test Sets

**Figure 1:**
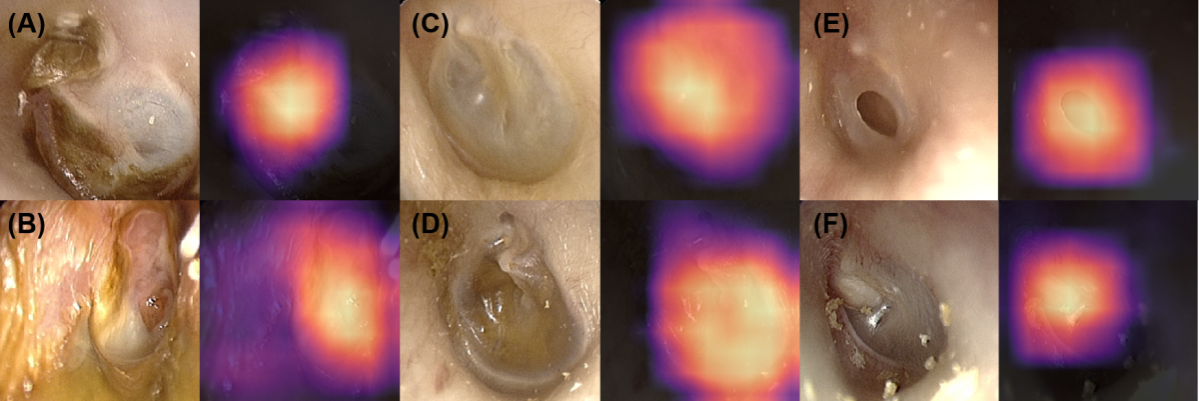
Representative class and their activation heatmap (Grad-CAM): (A) attic retraction), (B) myringitis or otitis externa, (C) normal findings, (D) otitis media with effusion, (E) tympanic perforation, (F) middle ear or external ear canal tumors.

We used a total of 6900 otoendoscopic images from 6 classes for training (Table 1). The training dataset was imbalanced, reflecting the prevalence of ear disease. Although the dataset was obtained based on a tertiary referral center, therefore having rich pathologic cases, normal classes were substantially common. The testing environment consisted of 2 different settings: (1) balanced test set (300 sample images), consisting of 6 classes with 50 images each, without considering the prevalence of ear diseases and (2) imbalanced test set (300 samples), each class distributed proportionally to the training dataset. Figure 1 displays representative classes and their activation heatmaps. The classification system could focus on important areas of eardrums and EACs. For attic retraction, the DL model focused on pathologic attic areas of the eardrum. When EACs were wet due to inflammation of the middle or external ear, it was visible in the heatmap. Normal and middle ear effusions have the same area of interest, mainly the eardrum and the middle ear cavity, which was correctly depicted by the classification system. Perforation of the tympanic membrane was visualized by the heatmap, as well as middle ear tumors inside the tympanic membrane (Figure 1).

### ML Model Performance Over Different Numbers of Training Samples, the Class Imbalance Problem, and Modifications

**Figure 2:**
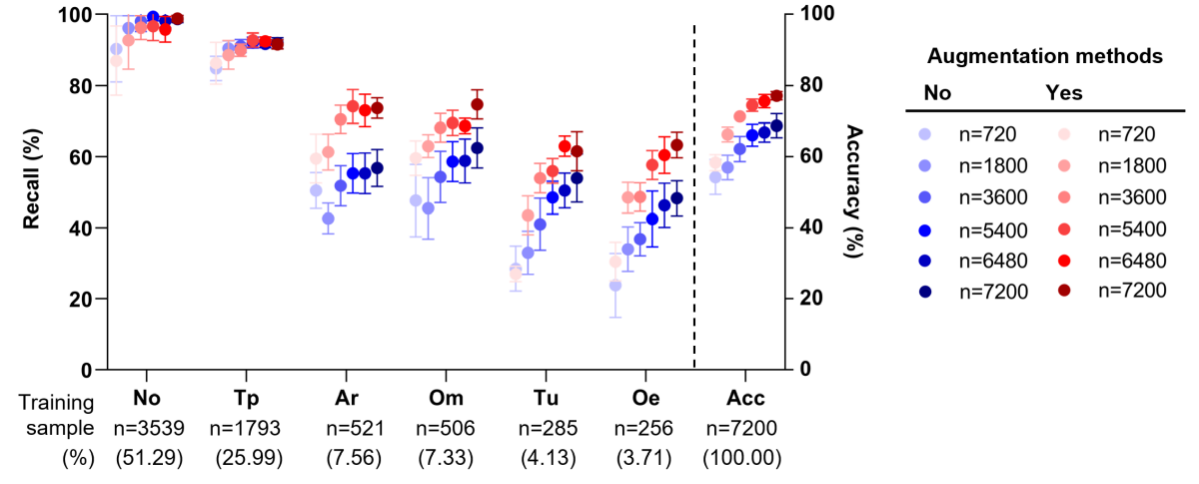
Per-class recall and overall classification accuracy (bars = 95% CI) for classes according to the number of training samples and augmentation, trained with 12 different convolutional neural network models and tested on the balanced test set. Acc: overall accuracy; Ar: attic retraction, destruction; No: normal; Oe: myringitis or acute otitis externa; Om: otitis media with effusion; Tp: tympanic perforation; Tu: middle or external ear canal tumors or cerumen impaction.

When testing with the baseline model (without adjustment of class imbalance in training), the overall average accuracy was 82.78% in the imbalanced (according to disease prevalence) test set. However, in the balanced test set, the overall accuracy was 68.69% (chance level: 16.7%), substantially inferior to the accuracy of 82.78% for the imbalanced testing data. To mitigate the class imbalance problem, we retrained a classification model using oversampling, mixup, and focal loss. We tested every combination of these strategies under the balanced testing environment. Applying all 3 strategies in the training phase had a synergistic effect, achieving an average of 8.41% gain (average accuracy: 77.14% vs 68.69%) in the balanced test set while compromising 0.75% in the imbalanced test set. Especially, oversampling was universally beneficial (Multimedia Appendix 2). The augmented classifier gained more per-class accuracy for classes with fewer samples, such as attic retractions, than the baseline model, leading to better overall accuracy in the balanced test set (n=7200; Figure 2; additional example results of both test sets with a Resnet101-based classifier available in Multimedia Appendix 3).

To explore the relationship between the classification bias and the size of the training dataset in detail, we compared the classification performance over different numbers of training samples when tested in a balanced testing environment. The overall accuracy increased with a higher number of samples. The adjustment for the class imbalance during the training steps improved the performance, particularly for classes with fewer training samples (Figure 2). For classes with a high incidence rate, there were no significant gains by augmentation as they already reached a plateau of accuracy, not to mention the oversampling method synthesizes more images for smaller classes to match the most common, “normal,” class. Nevertheless, augmenting images (affine transformations) for rare classes did not yet reach a saturated accuracy as the number of total training samples increased.

### AI Versus HI in Per-Class Accuracy and Interrater Variability

**Figure 3:**
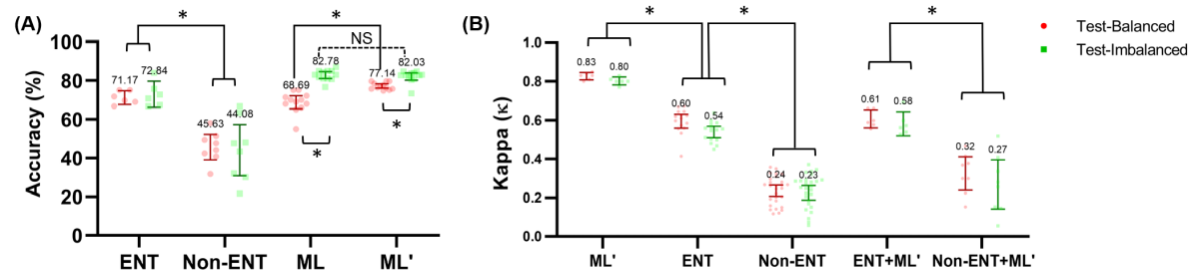
Mean (A) overall diagnostic accuracy and (B) Fleiss generalized kappa for interrater reliability (error bars = 95% CI); the predictions by the ResNet152-based deep learning model were assumed to be a human rater. ENT: otolaryngologists; ENT+ML': machine learning model plus otolaryngologists; ML: baseline machine learning models; ML': augmented machine learning models; Non-ENT: nonotolaryngologists; Non-ENT+ML': machine learning model plus nonotolaryngologists; NS: not statistically significant. *P<.001 (Mann-Whitney test: ENT vs Non-ENT; Wilcoxon matched-pairs signed-rank test: ML vs ML').

The diagnostic accuracy of the 2 test sets was evaluated separately (Table S2 in Multimedia Appendix 4; additional metrics including precision, recall, and F1 scores are in Multimedia Appendix 4). All participants, including prediction models, assessed the same collection of images in the same order to rule out bias caused by different questionnaire layouts. Otolaryngologists (n=6) significantly outperformed nonotolaryngologists (n=8) in both balanced (mean 71.17%, SD 3.37% vs mean 45.63%, SD 7.90%; Mann-Whitney U=0; *P*<.001) and imbalanced (mean 72.84%, SD 6.41% vs mean 44.08%, SD 15.84%; Mann-Whitney U=0.5; *P*=.001) test sets. Our fine-tuned CNN-based ML models (n=12) tended to be better than otolaryngologists (n=6) in both imbalanced (mean 82.03%, SD 3.06% vs mean 72.84, SD 6.41%; Mann-Whitney U=10.50; *P*=.014) and balanced (mean 77.14%, SD 1.84% vs mean 71.17%, SD 3.37%; Mann-Whitney U=3; *P*<.001) test sets and outperformed nonotolaryngologists in both test sets (Figure 3A).

Compared with nonotolaryngologists, ML models had better accuracy in all classes. Compared with otolaryngologists, ML models were better at predicting normal ears, tympanic perforations, and attic retractions, which were more prevalent in the training dataset. The diagnosis rate of otitis media with effusion and myringitis was similar between prediction models and otolaryngologists. For classifying tumorous conditions, otolaryngologists were better than prediction models in the balanced test set (Table S2 in Multimedia Appendix 4). The overall accuracy for all physicians was not significantly different between the balanced and imbalanced test sets, while both augmented (n=12; median 5.3; *P*=.001; Wilcoxon matched-pairs signed-rank test) and baseline (n=12; median 13.3; *P*<.001; Wilcoxon matched-pairs signed-rank test) ML models had significantly higher accuracy in the imbalanced test set (Figure 3A). Of note, augmented ML models had gained significant accuracy in the balanced test set (n=12; median 8.3; *P*<.001; Wilcoxon matched-pairs signed-rank test) without loss of accuracy in the imbalanced test set (n=12; median 0.8; *P*=.28; Wilcoxon matched-pairs signed-rank test) compared with ML models without augmentation.

Regarding variance in accuracy, ML models had similar prediction results across different models, resulting in a low SD (1.76%), which was much lower than that of the otolaryngology specialists (5.86%) and nonotolaryngologists (14.82%). The results of the Fleiss generalized kappa as a measure of interrater reliability are presented in Figure 3B. Between ML models, ĸ scores ranged between 0.77 and 0.85, indicating a substantial diagnostic similarity among ML models. The ĸ score was >0.60 between 2 otolaryngology specialists and mostly >0.50 between all otolaryngology specialists and residents, which corresponds to moderate agreement between them. However, it was mostly <0.30 between nonotolaryngologists, which may be interpreted as fair agreement between these physicians. The predictions by the ML models were more likely to resemble those of otolaryngologists than nonotolaryngologists, showing similarity to otolaryngologists (Figure 3B; ĸ=0.5947, SD 0.05, n=12 vs ĸ=0.2966, SD 0.13, n=16; *P*<.001; Mann-Whitney U test).

Using the 4 top-performing models (ResNet152, DPN92, InceptionV4, and Densenet201), we constructed an ensemble classifier by adding and taking the maximum arguments following the softmax activation function in each classifier. Using this approach, we were able to gain an average of 1.83% in the balanced dataset and 3.5% in the imbalanced dataset, reaching 80.33% and 86.67% overall accuracy, respectively (Table S2 in Multimedia Appendix 4). The ensemble classifier of the different models outperformed any other CNN-based classifier alone in overall accuracy and proved to be a stable model for final prediction. Indeed, ensembling had a positive but, at the same time, limited effect in enhancing the overall accuracy because of diagnostic similarity, as indicated by high ĸ scores between models.

### AI Compared With HI in Class Prevalence and Size of the Training Dataset

**Figure 4:**
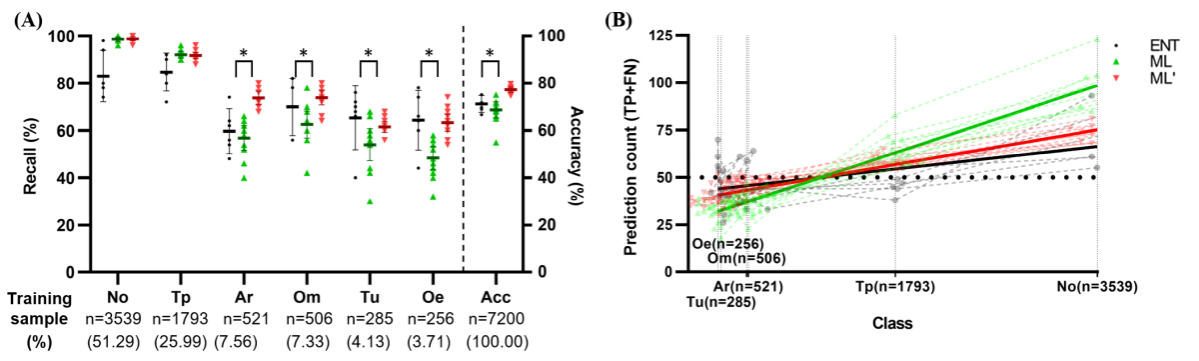
In the balanced test set, (A) per-class recall and overall accuracy (bars indicate 95% CI) and (B) prediction counts in individual classes (the dotted line at 50 indicates the sample size of the balanced test set for each class; x axis is on a logarithmic scale). Classes are listed left to right by descending number of training samples. Each class had 50 samples in the balanced test set (a total of 300 samples for all 6 classes). Nonotolaryngologists had too high variations and low accuracies and were not plotted. ENT: Y intercept=42.14 (95% CI 39.14-45.24), slope=0.006836 (95% CI 0.004805-0.008939), pseudo R-squared=0.3262; ML’: Y intercept=37.89 (95% CI 35.77-40.07), slope=0.01053 (95% CI 0.008981-0.01211), pseudo R-squared=0.8665; ML: Y intercept=26.68 (95% CI 24.73-28.69), slope=0.02028 (95% CI 0. 01861-0.02198), pseudo R-squared=0.9167. Acc: overall accuracy; Ar: attic retraction; ENT: otolaryngologist; FN: false negative; ML: baseline machine learning models; ML': augmented machine learning models; No: normal; Oe: myringitis or acute otitis externa; Om: otitis media with effusion; Tp: tympanic perforation; TP: true positive; Tu: middle or external ear canal tumors or cerumen impaction. *P<.01 (Wilcoxon matched-pairs signed rank test).

In otolaryngologists, accuracies tended to be stable regardless of sample sizes, whereas ML models showed a bias towards prevalent classes (Figure 4). Also, the augmentation method showed significantly improved accuracies in minor classes (attic retraction: n=12, median 15.0, *P*<.001; otitis media with effusion: n=12, median 13.0, *P*=.005; middle or external ear canal tumors or cerumen impaction: n=12, median 6.0, *P*=0.01; myringitis or acute otitis externa: n=12, median 13.0, *P*<.001; Wilcoxon matched-pairs signed rank tests). Otolaryngologists had a higher variance in the accuracies compared with the augmented ML models in prevalent classes (normal, tympanic perforation) and overall accuracy. We additionally analyzed the count of predicted samples, which corresponds to true-positive and false-negative predictions, for each class of the balanced test set. Each classification had 50 occurrences in the set, so ideally, the count of predicted samples (true positives and false negatives) should be 50, which is drawn as the dotted line in Figure 4B.

ML models showed more bias towards the number of training data, as more prevalent classes tended to have more than 50 counts (above the dotted line; normal class was above the line and therefore overly diagnosed), while rarer classes such as myringitis or acute otitis externa had a lower count (below the dotted line; underdiagnosed). Different classification tendencies of humans and machines were evaluated with respect to their dependency on class prevalence. The Poisson regression analysis for the correlation between each class’s number with the corresponding number of predictions showed a significantly different slope between otolaryngologists, augmented ML models, and baseline ML models (Figure 4B; slope: 0.021 for ML, 0.011 for ML’, and 0.007 for otolaryngologists). The likelihood ratio test with the null hypothesis had one curve for all data sets, and the alternative hypothesis had a different curve for each data set. The likelihood ratio between baseline ML models and the augmented ML models was 76.36 (*P*<.001), and the likelihood ratio between the augmented ML models and humans was 7.958 (*P*=.019). Differing slopes indicated that the ML models tended to produce more likely predictions based on the number of training samples.

Of note, otolaryngologists’ predictions were not well fitted linearly because of individual differences in prediction (pseudo R-squared=0.3262). While the augmented ML model had mitigated the class imbalance problems, it still preferred prevalent classes, which was not apparent with otolaryngologists.

## Discussion

### Principal Findings

The main implications of this study are 3-fold: (1) Work by HI and AI shows different behaviors (prevalence dependency and interrater variability); (2) data augmentation reduces the class imbalance problem but the result is different according to the sample sizes of each class, requiring a certain amount of data samples for the rare class to achieve a reliable level; and (3) considering the high accuracy comparable to otologists and high variations in diagnostic performance by site clinicians, our ML model may act as a virtual otoendoscopic image analysis consultant, as long as clinicians consider that this ML model considers only images and there are potential biases in the ML models toward prevalence.

First, we showed that machines work in different ways than human knowledge, which is exemplarily reflected in the effects of class imbalance. As expected, ML models showed bias toward higher prevalent samples in the training set, but lower interrater (or ML model) variations. In contrast, human experts showed high interrater variations in their classifications but no prevalence-dependent biases. For example, the normal class is diagnosed when all other pathologies are excluded; hence, it is inherently difficult to diagnose despite its extensive prevalence. Meanwhile, cerumen impaction and tympanic perforation were less prevalent in the dataset, but they were classified correctly more times than the normal class by the human raters because of the obvious findings. Attic retractions and otitis media with effusions were subtle in many cases; hence, they were diagnosed with lower accuracy (Figure 4A). Therefore, for physicians, the difficulty lies mainly in class-specific abstract rules, which the data-driven ML model does not detect.

Second, although the class imbalance problem was mitigated by combining strategies in the training phase (oversampling, mixup, and focal loss), it had less effect for prevalent diseases but more for rare diseases (Figure 2). For the data-driven approach using ML, finding the hyperspace of features that covers within-class diversity, different from the other classes, is not trivial. ML attempts to find within-class diversity using imaging features based on statistics, which demands a large sample size to capture within-class variability. Indeed, in ML models, a higher number of samples in training produced better accuracies and reduced model variability (Figure 2), which is in line with the results of a previous study [9]. In reality, due to low incidence, we lacked a sufficient number of data samples for less prevalent diseases. Data augmentation improves the overall accuracy and recall of individual classes, especially for less prevalent classes. However, data augmentation was performed by manipulating the given dataset, which limited its diversity within images for rare classes compared with that of prevalent classes. Therefore, having more actual data for training is still essential for higher performance, particularly for rare classes. Often, datasets contain an abundance of normal and common disease classes and lack uncommon diseases. It is a general problem in the field of medical imaging, especially when diseases are rare and obtaining sufficient samples is difficult [35].

Third, our ML model showed the possibility of acting as a physician’s assistant in real-world clinics. Inconsistent performance in humans was apparent, especially in the group of nonotolaryngologists (ĸ=0.24, 95% CI 0.21-0.26) compared with ML models (ĸ=0.83, 95% CI 0.81-0.84). Physicians often overestimated their skills despite the variance in their diagnostic capabilities, leading to faulty and inconsistent clinical information delivered to patients. Meanwhile, machines sometimes produced errors in trivial cases, even if their overall accuracies were expected to be on par or better than those of otolaryngologists. When making diagnostic suggestions, physicians’ decisions should be taken into account to compensate for faulty ML suggestions, not to mention that the final responsibility of the decision should be on the care provider. In a previous study, diagnosis of middle ear disease by nonspecialists was reportedly only 30% in a study with primary care trainees [36] and 50% in a study with pediatricians just after finishing a continuing medical examination course [37]. Even for otolaryngologists, the accuracy of diagnosing otitis media using a pneumatic otoscope was 73% [37], which implies that accurate diagnosis using otoscopy is challenging [13,14,17]. Computer-aided diagnosis may be beneficial for both experts and nonotolaryngologists, for example, with our proposed ML model.

It is worth mentioning that our ML model acted as an otolaryngologist since the interrater variability (kappa) score between the ML model and otolaryngologist was similar to the kappa score between otolaryngologists (Figure 3B, ENT and ENT+ML’). Therefore, having our ML models interpret otoendoscopic images may be similar to having an on-demand otolaryngology consultant. Considering the shortage of specialists, nonotolaryngologists may combine our image interpretation results and clinical manifestations, which our ML does not consider, to deliver an accurate diagnosis and care for their patients.

### Limitations

We point out the limitations and future directions of our study. Due to privacy issues, we could not perform our model outside the institution, and external validation could not be performed. However, our otoendoscopic images were acquired from a diverse set of types of imaging equipment, which may mimic external validation. Also, as pointed out in our Methods section, one image may have multiple pathologies but was labeled according to the labeling priority. Multilabel classification should be conducted in the future, along with multimodal models that consider a patient’s clinical information. Last but not least, although our ML models showed good accuracy in analyzing images, the current model does not consider additional clinical information, which most clinicians consider when making a diagnosis. Therefore, our ML model’s higher accuracy in image translation may not necessarily correlate to better diagnostic expertise to physicians in the real world.

### Comparison With Prior Work

In our previous study [9], we also classified ear disease into 6 entities but tested our model in a 5-fold cross-validation manner. Therefore, overall accuracy was less affected by classes of lower prevalence, showing inferior performance when applying the model in real-world clinics. A more recent study by Byun et al [38] assessed the effects of diagnostic assistant systems when used by otolaryngology residents. However, the diversity of disease was limited (only 4 diseases) and did not cover all ear diseases, especially external ear diseases and tumors. Also, the test set’s size was small and did not test under various circumstances, that is balanced and imbalanced test sets. Our work addressed these effects and tests in both settings with a larger test set and more importantly, nonotolaryngologists, who may benefit most from using diagnostic assistance. We also measured the interrater reliability using kappa statistics, proving our proposed ML model similar to an otolaryngologist rather than a general practitioner.

### Conclusions

Among the many potential differences, we focused on the data-driven classification bias of AI due to class imbalances of data in real-world clinics. ML is trained to find statistically optimal features from a large amount of training data in a way that improves the overall classification accuracy. Different numbers of samples in different classes due to imbalanced incidences inherent in the human population induce difficulty in building a reliable ML model. Based on the results of class imbalance, sample size, and accuracy (Figure 2), we still prefer a large but imbalanced dataset to a small but balanced dataset for a robust ML model. Therefore, our future system should analyze the strengths and weaknesses of the human experts and weigh the ML results to make suggestions depending on the situation: It provides strong suggestions when ML is superior and weak suggestions when ML is vulnerable. Along with suggestions, the system may display relative confidence in its diagnostic ability. Especially in atypical and rare diseases, this approach may provide more robust diagnoses, making the prediction system similar to consulting a fellow expert trained in a different institution for a second opinion.

Considering the practical situation in the clinical field that is short of otolaryngology specialists, clinicians may utilize our diagnostic assistance systems to deliver reliable patient care, while keeping in mind that the ML model does not consider additional clinical information and could be biased toward prevalent diseases.

## Appendix

Multimedia Appendix 1 Mixup strategy for oversampling and focal loss for loss function.

Multimedia Appendix 2 Effects of augmentation techniques applied to classification models.

Multimedia Appendix 3 Confusion matrix in imbalanced and balanced test set.

Multimedia Appendix 4 Supplementary tables.

## Abbreviations

ADAM: Adaptive Moment Estimation
AI: artificial intelligence
CNN: convolutional neural network
DL: deep learning
EAC: external auditory canal
HI: human intelligence
ML: machine learning
