# Supplementary material for: Differential Biases and Variabilities of Deep Learning–Based Artificial Intelligence and Human Experts in Clinical Diagnosis: Retrospective Cohort and Survey Study
Source: JMIR Med Inform. 2021 Dec 8;9(12):e33049. doi: 10.2196/33049 (PMC8701703; doi:10.2196/33049)
Supplement: Multimedia Appendix 1 [file medinform_v9i12e33049_app1.docx]

**Mixup strategy for oversampling**

Mixup strategy [30] is to make virtual feature-target vectors using two previously defined features. The strategy binds two different classifications to one classification using such that:

$$\tilde{x}=tx_{i}+\left( 1-t \right)x_{j}$$

$$\tilde{y}=ty_{i}+\left( 1-t \right)y_{j}$$

, where $\left( x_{i},x_{j} \right)$ refers to image 1 and 2, and $\left( y_{i},y_{j} \right)$ refers to the labels of images 1 and 2 and t is a random number between 0 and 1. For example, we arbitrarily create an image that is 30% tympanic perforation and 70% otitis externa and train the system using the newly created label.

**Focal loss for loss function**

The loss function of training was modified. Usually, for image classification, the cross-entropy loss is used as a loss function. In short notation, the cross-entropy loss is defined as:

$$\mathrm{CE}\left( p_{t} \right)= -\log\left( p_{t} \right)$$

, where $p_{t}$ is defined as $p$ if $y=1$, or $1-p$ if otherwise, and $p$ represents the model’s probability for the class labeled $y$. Lin et al. proposed modification to this loss function [31], known as focal loss, which modulates the cross-entropy loss with a factor as

$$\mathrm{FL}\left( p_{t} \right)= -\left( 1-p_{t} \right)^{\gamma}\log\left( p_{t} \right)$$

where γ ≥ 0. When γ = 0, focal loss is identical to cross-entropy loss. The formula $\left( 1-p_{t} \right)^{\gamma}$ is the modulating factor of focal loss that has a boosting effect of misclassified training images by setting the γ (focusing parameter) value when training. We tested γ = 1, 2, and 4 and chose 1 as the focusing parameter, which yielded stable accuracy across different CNN-based models.
