# Supplementary material for: Differential Biases and Variabilities of Deep Learning–Based Artificial Intelligence and Human Experts in Clinical Diagnosis: Retrospective Cohort and Survey Study
Source: JMIR Med Inform. 2021 Dec 8;9(12):e33049. doi: 10.2196/33049 (PMC8701703; doi:10.2196/33049)
Supplement: Multimedia Appendix 2 [file medinform_v9i12e33049_app2.docx]

*
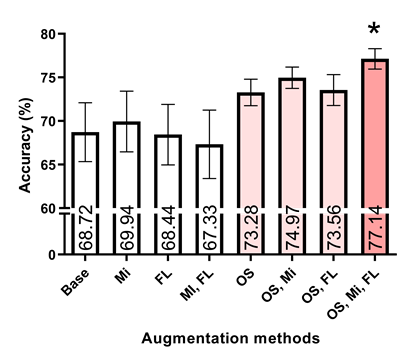
*

**Figure. S1.** Effects of augmentation techniques applied to classification models .

Gain of accuracy with various modification methods. Accuracy is measured on the balanced test set.

Mi: Mixup, FL: Focal Loss, OS: Oversampling

Accuracy of Base model: 68.72 ± 5.308 (95% Confidence interval: 65.35 to 72.09)

Accuracy of Augmented (OS, Mi, FL) model: 77.14 ± 1.839 (95% Confidence interval: 75.97 to 78.31)

*: Statistically significant (p<0.0001) [Repeated measures one-way ANOVA]
