# Supplementary material for: Differential Biases and Variabilities of Deep Learning–Based Artificial Intelligence and Human Experts in Clinical Diagnosis: Retrospective Cohort and Survey Study
Source: JMIR Med Inform. 2021 Dec 8;9(12):e33049. doi: 10.2196/33049 (PMC8701703; doi:10.2196/33049)
Supplement: Multimedia Appendix 3 [file medinform_v9i12e33049_app3.docx]

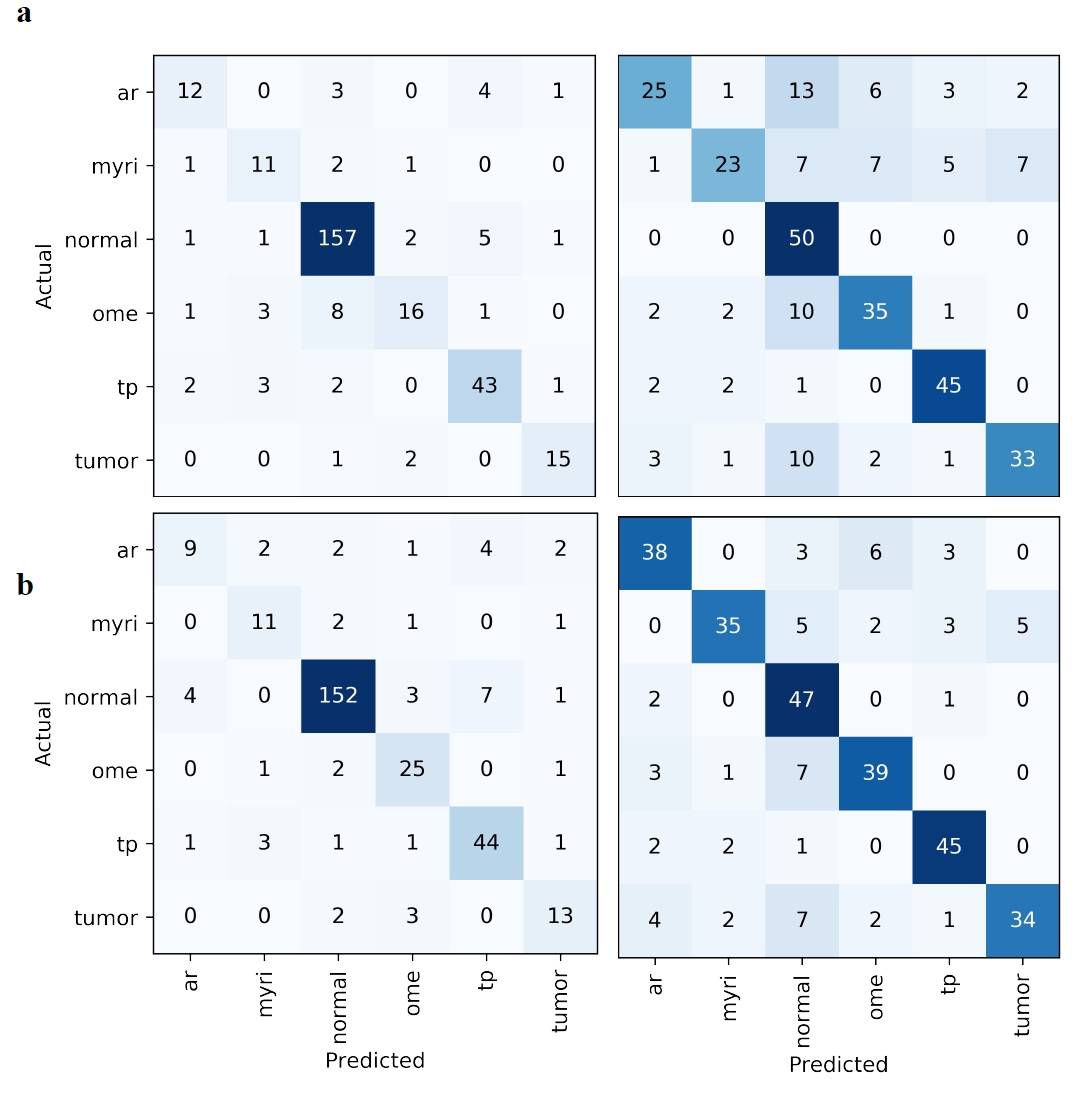


**Figure S2.** Confusion matrix in imbalanced and balanced test set

**(a)** Confusion matrix of a baseline Resnet101-based classifier in imbalanced (left) and balanced (right) testing environment. Accuracy is 72% and 85%, respectively.

**(b)** Confusion matrix of a Resnet101 classifier with augmentation in imbalanced (left) and balanced (right) environment. Accuracy is 79% and 83%, respectively.
