# Supplementary material for: Differential Biases and Variabilities of Deep Learning–Based Artificial Intelligence and Human Experts in Clinical Diagnosis: Retrospective Cohort and Survey Study
Source: JMIR Med Inform. 2021 Dec 8;9(12):e33049. doi: 10.2196/33049 (PMC8701703; doi:10.2196/33049)
Supplement: Multimedia Appendix 4 [file medinform_v9i12e33049_app4.docx]

**Table S1**. Performance table of training models without additional strategies for class imbalance.

| Transferred Models | Parameters  (millions) | Mean Accuracy (%) | | | |
| --- | --- | --- | --- | --- | --- |
|  |  | N=720 | N=1,800 | N=3,600 | N=6,480 |
| VGG19 | 20.5 | 52.27 | 57.40 | 63.47 | 67.60 |
| ResNet101 | 44.6 | 58.27 | 65.00 | 69.00 | 73.13 |
| ResNet152 | 60.2 | 60.27 | 65.27 | 69.47 | 72.33 |
| SeNet154 | 115.1 | 58.67 | 60.60 | 63.80 | 68.13 |
| DPN92 | 37.7 | 57.80 | 63.13 | 67.73 | 73.40 |
| Xception | 22.9 | 37.07 | 44.00 | 51.47 | 63.73 |
| InceptionV3 | 23.8 | 50.40 | 53.47 | 58.47 | 65.13 |
| InceptionResNetV2 | 55.8 | 51.00 | 53.40 | 61.33 | 65.53 |
| NASNetAMobile | 5.3 | 45.40 | 47.47 | 51.53 | 55.13 |
| PNASNetLarge | 86.1 | 51.13 | 55.87 | 60.47 | 67.20 |
| InceptionV4 | 42.7 | 49.20 | 49.80 | 57.67 | 63.93 |
| Densenet201 | 20.0 | 58.00 | 61.40 | 66.33 | 70.80 |
| Mean | - | 52.46 | 56.40 | 61.73 | *67.17 |
| Mean SD | - | 6.66 | 6.82 | 6.01 | *5.07 |

Accuracy was measured in the balanced dataset.

Images were selected randomly by using different random seeds and each model was trained five times to rule out training bias.

N : the number of images in the training set.

*: Statistically significant (p=0.0079,Mann-Whitney test)

Table S2. Comparison of the accuracy between humans and the machine learning (ML) models.

| Reviewer | Per-class recall (correctness) | | | | | | | | | | | | Accuracy | |
| --- | --- | --- | --- | --- | --- | --- | --- | --- | --- | --- | --- | --- | --- | --- |
|  | No^a^ | | Tp^d^ | | Ar^e^ | | Oe^f^ | | Om^g^ | | Tu^h^ | |  |  |
|  | T_Bal_^b^ | T_Imbal_^c^ | T_Bal_ | T_Imbal_ | T_Bal_ | T_Imbal_ | T_Bal_ | T_Imbal_ | T_Bal_ | T_Imbal_ | T_Bal_ | T_Imbal_ | T_Bal_ | T_Imbal_ |
| **Otolaryngologists, %** | | | | | | | | | | | | | | |
| ENT^i^-1 | 74.00 | 91.62 | 90.00 | 92.16 | 62.00 | 50.00 | 66.00 | 46.67 | 82.00 | 75.86 | 76.00 | 72.22 | 75.00 | 84.00 |
| ENT-2 | 78.00 | 82.63 | 92.00 | 98.04 | 54.00 | 25.00 | 44.00 | 20.00 | 78.00 | 68.97 | 72.00 | 61.11 | 69.67 | 75.67 |
| ENT-R^j^4 | 98.00 | 76.65 | 90.00 | 82.35 | 56.00 | 50.00 | 60.00 | 66.67 | 56.00 | 55.17 | 70.00 | 66.67 | 71.67 | 70.67 |
| ENT-R3 | 80.00 | 71.26 | 84.00 | 80.39 | 64.00 | 75.00 | 78.00 | 66.67 | 78.00 | 58.62 | 66.00 | 55.56 | 75.00 | 72.67 |
| ENT-R2 | 94.00 | 76.65 | 72.00 | 52.94 | 74.00 | 55.00 | 64.00 | 40.00 | 70.00 | 75.86 | 40.00 | 44.44 | 69.00 | 66.67 |
| ENT-R1 | 74.00 | 71.26 | 80.00 | 72.55 | 48.00 | 55.00 | 74.00 | 66.67 | 56.00 | 37.93 | 68.00 | 66.67 | 66.67 | 67.33 |
| All-ENT, %(SD) | 83.00  (10.41) | 78.34 (7.76) | 84.67 (7.65) | 79.74 (15.91) | 59.67 (9.07) | 51.67 (16.03) | 64.33 (11.96) | 51.11 (19.17) | 70.00 (11.52) | 62.07 (14.63) | 65.33 (12.87) | 61.11 (9.94) | 71.17 (3.37) | 72.83 (6.41) |
| **Nonotolaryngologists, %** | | | | | | | | | | | | | | |
| FM^k^-1 | 80.00 | 75.45 | 64.00 | 76.47 | 30.00 | 35.00 | 44.00 | 33.33 | 70.00 | 41.38 | 60.00 | 61.11 | 58.00 | 66.67 |
| FM-2 | 54.00 | 44.91 | 76.00 | 52.94 | 34.00 | 25.00 | 50.00 | 53.33 | 28.00 | 24.14 | 54.00 | 44.44 | 49.33 | 43.33 |
| EM^l^-1 | 60.00 | 19.16 | 58.00 | 54.90 | 48.00 | 35.00 | 4.00 | 26.67 | 16.00 | 20.69 | 78.00 | 72.22 | 44.00 | 30.00 |
| EM-2 | 54.00 | 54.49 | 22.00 | 31.37 | 40.00 | 55.00 | 76.00 | 66.67 | 50.00 | 41.38 | 12.00 | 16.67 | 42.33 | 47.67 |
| GP^m^-1 | 18.00 | 9.58 | 46.00 | 50.98 | 40.00 | 25.00 | 28.00 | 26.67 | 34.00 | 41.38 | 24.00 | 11.11 | 31.67 | 21.67 |
| GP-2 | 44.00 | 22.75 | 66.00 | 52.94 | 44.00 | 25.00 | 18.00 | 46.67 | 24.00 | 27.59 | 48.00 | 61.11 | 40.67 | 32.00 |
| GP-3 | 84.00 | 81.44 | 56.00 | 50.98 | 24.00 | 50.00 | 44.00 | 40.00 | 50.00 | 31.03 | 28.00 | 16.67 | 47.67 | 63.33 |
| GP-4 | 64.00 | 47.90 | 66.00 | 62.75 | 20.00 | 35.00 | 40.00 | 20.00 | 44.00 | 41.38 | 74.00 | 55.56 | 51.33 | 48.00 |
| Non-ENTs, %(SD) | 57.25 (20.78) | 44.46 (26.10) | 56.75 (16.56) | 54.17 (12.61) | 35.00 (9.80) | 35.63 (11.48) | 38.00 (21.78) | 39.17 (15.71) | 39.50 (17.43) | 33.62 (8.79) | 47.25 (23.97) | 42.36 (24.11) | 45.63 (7.89) | 44.08 (15.83) |
| Physicians, %(SD) | 68.29 (21.19) | 58.98 (26.31) | 68.71 (19.38) | 65.13 (18.85) | 45.58 (15.61) | 42.50 (15.41) | 46.52 (22.21) | 44.29 (17.65) | 52.57 (21.45) | 45.81 (18.37) | 65.33 (21.44) | 50.40 (21.07) | 56.57 (14.49) | 56.40 (19.21) |
| **ML Models** | | | | | | | | | | | | | | |
| VGG19 | 98.00 | 89.22 | 90.00 | 86.27 | 76.00 | 55.00 | 56.00 | 80.00 | 72.00 | 82.76 | 62.00 | 61.11 | 75.67 | 83.67 |
| ResNet101 | 100.00 | 85.03 | 94.00 | 84.31 | 78.00 | 65.00 | 62.00 | 53.33 | 76.00 | 72.41 | 68.00 | 66.67 | 79.67 | 79.67 |
| ResNet152 | 98.00 | 92.22 | 92.00 | 86.27 | 72.00 | 65.00 | 74.00 | 80.00 | 72.00 | 65.52 | 62.00 | 66.67 | 78.33 | 84.67 |
| SeNet154 | 98.00 | 92.22 | 92.00 | 86.27 | 68.00 | 45.00 | 66.00 | 66.67 | 80.00 | 75.86 | 62.00 | 72.22 | 77.67 | 84.00 |
| DPN92 | 98.00 | 91.02 | 92.00 | 86.27 | 80.00 | 40.00 | 64.00 | 73.33 | 78.00 | 75.86 | 66.00 | 61.11 | 79.67 | 82.67 |
| Xception | 100.00 | 83.83 | 92.00 | 82.35 | 72.00 | 65.00 | 64.00 | 73.33 | 78.00 | 79.31 | 56.00 | 66.67 | 77.00 | 80.33 |
| InceptionV3 | 100.00 | 88.62 | 92.00 | 88.24 | 68.00 | 60.00 | 60.00 | 86.67 | 78.00 | 72.41 | 60.00 | 38.89 | 76.33 | 82.00 |
| InceptionResnetV2 | 98.00 | 86.83 | 88.00 | 90.20 | 72.00 | 60.00 | 60.00 | 66.67 | 72.00 | 65.52 | 64.00 | 55.56 | 75.67 | 80.67 |
| NASNetAMobile | 96.00 | 77.84 | 92.00 | 78.43 | 70.00 | 55.00 | 68.00 | 53.33 | 64.00 | 65.52 | 60.00 | 66.67 | 75.00 | 73.33 |
| PNASNetLarge | 100.00 | 91.62 | 90.00 | 84.31 | 70.00 | 60.00 | 54.00 | 73.33 | 74.00 | 79.31 | 60.00 | 66.67 | 74.67 | 84.67 |
| InceptionV4 | 100.00 | 87.43 | 96.00 | 86.27 | 80.00 | 60.00 | 70.00 | 80.00 | 76.00 | 82.76 | 56.00 | 55.56 | 79.67 | 82.67 |
| Densenet201 | 100.00 | 89.22 | 90.00 | 86.27 | 78.00 | 50.00 | 62.00 | 73.33 | 66.00 | 79.31 | 62.00 | 61.11 | 76.33 | 82.67 |
| Ensemble^n^ | 100.0 | 92.81 | 94.00 | 86.27 | 76.00 | 60.00 | 76.00 | 80.00 | 74.00 | 79.32 | 64.00 | 77.78 | 80.33 | 86.67 |
| CNNs^o^, %(SD) | 98.83 (1.34) | 87.92 (4.17) | 91.67 (2.06) | 85.46 (2.95) | 73.67 (4.50) | 56.67 (8.07) | 63.33 (5.68) | 71.67 (10.29) | 73.83 (4.93) | 74.71 (6.47) | 61.50 (3.52) | 61.57 (8.69) | 77.14 (1.83) | 82.03 (3.06) |

^a^No: normal.

^b^T_Bal_: balanced test set.

^c^T_Imbal_: imbalanced test set.

^d^Tp: tympanic perforation.

^e^Ar: attic retraction.

^f^Oe: myringitis or acute otitis externa.

^g^Om: otitis media with effusion.

^h^Tu: middle or external ear canal tumors or cerumen impaction.

^i^ENT: otolaryngologist.

^j^ENT-R: otolaryngology resident (numbers indicate years of training).

^k^FM: family medicine specialist.

^l^EM: emergency medicine specialist.

^m^GP: general practitioner.

^n^Ensemble of ResNet152, DPN92, InceptionV4, and Densenet201 models**.**

^o^CNNs: convolutional neural networks.

Table S3. Comparison of human and machine learning model’s precision. Overall precision is macro-average precision.†Ensemble: Ensemble of ResNet152, DPN92, InceptionV4, Densenet201 model. Results not included to CNN statistics. T_Bal_: Balanced test set, T_Imbal_: Imbalanced test set. ENT: otolaryngologists; ENT-R: otolaryngology residents (numbers indicate years of training); FM: family medicine specialists; EM: emergency medicine specialists; GP: general practitioners. CNN: convolutional neural network. Other abbreviations are same as Figure 1

| Reviewer | Per-class precision | | | | | | | | | | | | Overall precision | |
| --- | --- | --- | --- | --- | --- | --- | --- | --- | --- | --- | --- | --- | --- | --- |
|  | No | | Tp | | Ar | | Oe | | Om | | Tu | |  |  |
|  | T_Bal_ | T_Imbal_ | T_Bal_ | T_Imbal_ | T_Bal_ | T_Imbal_ | T_Bal_ | T_Imbal_ | T_Bal_ | T_Imbal_ | T_Bal_ | T_Imbal_ | T_Bal_ | T_Imbal_ |
| ENT-1 | 60.66 | 89.47 | 95.74 | 92.16 | 72.09 | 83.33 | 82.50 | 53.85 | 67.21 | 59.46 | 79.17 | 81.25 | 76.23 | 76.59 |
| ENT-2 | 63.93 | 83.64 | 83.64 | 84.75 | 75.00 | 71.43 | 73.33 | 37.50 | 60.94 | 40.82 | 66.67 | 91.67 | 70.58 | 68.30 |
| ENT-R4 | 59.04 | 88.81 | 91.84 | 93.18 | 70.00 | 65.22 | 71.43 | 32.26 | 84.85 | 30.91 | 66.04 | 76.92 | 73.86 | 64.55 |
| ENT-R3 | 72.73 | 90.78 | 95.45 | 91.30 | 72.73 | 66.67 | 63.93 | 24.39 | 66.10 | 37.21 | 89.19 | 85.71 | 76.69 | 66.01 |
| ENT-R2 | 50.54 | 91.54 | 94.74 | 86.05 | 84.09 | 64.71 | 57.14 | 16.13 | 81.40 | 42.31 | 76.92 | 54.55 | 74.14 | 59.21 |
| ENT-R1 | 50.00 | 85.91 | 88.89 | 96.43 | 72.73 | 45.83 | 52.86 | 18.75 | 73.68 | 39.29 | 85.00 | 72.73 | 70.53 | 59.82 |
| FM-1 | 64.52 | 87.50 | 84.21 | 90.70 | 51.72 | 46.67 | 42.31 | 21.74 | 54.69 | 24.49 | 54.55 | 42.31 | 58.67 | 52.23 |
| FM-2 | 51.92 | 87.21 | 86.36 | 67.50 | 51.52 | 11.63 | 35.71 | 11.94 | 40.00 | 41.18 | 40.91 | 17.02 | 51.07 | 39.41 |
| EM-1 | 42.25 | 91.43 | 74.36 | 80.00 | 36.92 | 12.73 | 11.76 | 5.48 | 26.67 | 28.57 | 50.00 | 16.05 | 40.33 | 39.04 |
| EM-2 | 50.00 | 92.86 | 91.67 | 66.67 | 28.99 | 19.30 | 32.48 | 10.53 | 64.10 | 52.17 | 66.67 | 100.00 | 55.65 | 56.92 |
| GP-1 | 32.14 | 80.00 | 60.53 | 70.27 | 27.40 | 7.04 | 22.22 | 6.56 | 30.36 | 21.43 | 28.57 | 3.64 | 33.54 | 31.49 |
| GP-2 | 45.83 | 86.36 | 67.35 | 67.50 | 46.81 | 13.89 | 23.68 | 7.87 | 18.46 | 21.05 | 45.28 | 20.75 | 41.24 | 36.24 |
| GP-3 | 44.21 | 78.61 | 80.00 | 63.41 | 23.08 | 22.73 | 37.29 | 42.86 | 60.98 | 47.37 | 77.78 | 33.33 | 53.89 | 48.05 |
| GP-4 | 40.00 | 89.89 | 66.00 | 65.31 | 58.82 | 10.94 | 48.78 | 14.29 | 50.00 | 31.58 | 54.41 | 25.64 | 53.00 | 39.61 |
| VGG19 | 67.12 | 94.90 | 77.59 | 80.00 | 79.17 | 52.38 | 80.00 | 80.00 | 72.00 | 70.59 | 86.11 | 61.11 | 77.00 | 73.16 |
| ResNet101 | 65.79 | 92.81 | 81.03 | 69.35 | 86.67 | 68.42 | 88.57 | 57.14 | 86.36 | 65.63 | 80.95 | 60.00 | 81.56 | 68.89 |
| ResNet152 | 63.64 | 92.77 | 82.14 | 84.62 | 87.80 | 68.42 | 84.09 | 63.16 | 75.00 | 73.08 | 91.18 | 66.67 | 80.64 | 74.78 |
| SeNet154 | 66.22 | 91.12 | 80.70 | 75.86 | 72.34 | 69.23 | 89.19 | 62.50 | 83.33 | 81.48 | 83.78 | 76.47 | 79.26 | 76.11 |
| DPN92 | 71.01 | 94.41 | 80.70 | 74.58 | 80.00 | 80.00 | 86.49 | 57.89 | 81.25 | 66.67 | 84.62 | 61.11 | 80.68 | 72.44 |
| Xception | 72.46 | 93.96 | 80.70 | 64.62 | 75.00 | 61.90 | 78.05 | 73.33 | 75.00 | 79.31 | 84.85 | 57.14 | 77.68 | 71.71 |
| InceptionV3 | 67.57 | 92.50 | 80.70 | 73.77 | 80.95 | 70.59 | 78.95 | 59.09 | 78.00 | 70.00 | 76.92 | 70.00 | 77.18 | 72.66 |
| InceptionResnetV2 | 65.33 | 92.36 | 81.48 | 68.66 | 75.00 | 70.59 | 78.95 | 50.00 | 76.60 | 76.00 | 84.21 | 71.43 | 76.93 | 71.51 |
| NASNetAMobile | 70.59 | 91.55 | 80.70 | 62.50 | 76.09 | 44.00 | 72.34 | 36.36 | 72.73 | 73.08 | 78.95 | 57.14 | 75.23 | 60.77 |
| PNASNetLarge | 61.73 | 91.62 | 72.58 | 82.69 | 85.37 | 70.59 | 69.23 | 61.11 | 86.05 | 82.14 | 88.24 | 66.67 | 77.20 | 75.80 |
| InceptionV4 | 72.46 | 95.42 | 78.69 | 78.57 | 81.63 | 60.00 | 68.63 | 60.00 | 95.00 | 72.73 | 93.33 | 55.56 | 81.62 | 70.38 |
| Densenet201 | 64.10 | 93.13 | 69.23 | 75.86 | 86.67 | 66.67 | 86.11 | 57.89 | 84.62 | 79.31 | 83.78 | 57.89 | 79.09 | 71.79 |
| Ensemble† | 72.46 | 93.94 | 76.67 | 80.00 | 86.67 | 75.00 | 79.55 | 75.00 | 84.44 | 82.14 | 89.19 | 70.00 | 81.50 | 79.35 |

Table S4. Comparison of human and machine learning model’s recall. Overall recall is macro-average recall. †Ensemble: Ensemble of ResNet152, DPN92, InceptionV4, Densenet201 model. Results not included to CNN statistics. T_Bal_: Balanced test set, T_Imbal_: Imbalanced test set. ENT: otolaryngologists; ENT-R: otolaryngology residents (numbers indicate years of training); FM: family medicine specialists; EM: emergency medicine specialists; GP: general practitioners. CNN: convolutional neural network. Other abbreviations are same as Figure 1

| Reviewer | Per-class recall | | | | | | | | | | | | Overall recall | |
| --- | --- | --- | --- | --- | --- | --- | --- | --- | --- | --- | --- | --- | --- | --- |
|  | No | | Tp | | Ar | | Oe | | Om | | Tu | |  |  |
|  | T_Bal_ | T_Imbal_ | T_Bal_ | T_Imbal_ | T_Bal_ | T_Imbal_ | T_Bal_ | T_Imbal_ | T_Bal_ | T_Imbal_ | T_Bal_ | T_Imbal_ | T_Bal_ | T_Imbal_ |
| ENT-1 | 74.00 | 91.62 | 90.00 | 92.16 | 62.00 | 50.00 | 66.00 | 46.67 | 82.00 | 75.86 | 76.00 | 72.22 | 75.00 | 71.42 |
| ENT-2 | 78.00 | 82.63 | 92.00 | 98.04 | 54.00 | 25.00 | 44.00 | 20.00 | 78.00 | 68.97 | 72.00 | 61.11 | 69.67 | 59.29 |
| ENT-R4 | 98.00 | 76.65 | 90.00 | 82.35 | 56.00 | 75.00 | 60.00 | 66.67 | 56.00 | 55.17 | 70.00 | 66.67 | 71.67 | 67.92 |
| ENT-R3 | 80.00 | 71.26 | 84.00 | 80.39 | 64.00 | 50.00 | 78.00 | 66.67 | 78.00 | 58.62 | 66.00 | 55.56 | 75.00 | 66.25 |
| ENT-R2 | 94.00 | 76.65 | 72.00 | 52.94 | 74.00 | 55.00 | 64.00 | 40.00 | 70.00 | 75.86 | 40.00 | 44.44 | 69.00 | 61.68 |
| ENT-R1 | 74.00 | 71.26 | 80.00 | 72.55 | 48.00 | 55.00 | 74.00 | 66.67 | 56.00 | 37.93 | 68.00 | 66.67 | 66.67 | 57.48 |
| FM-1 | 80.00 | 75.45 | 64.00 | 76.47 | 30.00 | 35.00 | 44.00 | 33.33 | 70.00 | 41.38 | 60.00 | 61.11 | 58.00 | 53.79 |
| FM-2 | 54.00 | 44.91 | 76.00 | 52.94 | 34.00 | 25.00 | 50.00 | 53.33 | 28.00 | 24.14 | 54.00 | 44.44 | 49.33 | 40.79 |
| EM-1 | 60.00 | 19.16 | 58.00 | 54.90 | 48.00 | 35.00 | 4.00 | 26.67 | 16.00 | 20.69 | 78.00 | 72.22 | 44.00 | 38.11 |
| EM-2 | 54.00 | 54.49 | 22.00 | 31.37 | 40.00 | 55.00 | 76.00 | 66.67 | 50.00 | 41.38 | 12.00 | 16.67 | 42.33 | 44.26 |
| GP-1 | 18.00 | 9.58 | 46.00 | 50.98 | 40.00 | 25.00 | 28.00 | 26.67 | 34.00 | 41.38 | 24.00 | 11.11 | 31.67 | 27.45 |
| GP-2 | 44.00 | 22.75 | 66.00 | 52.94 | 44.00 | 25.00 | 18.00 | 46.67 | 24.00 | 27.59 | 48.00 | 61.11 | 40.67 | 39.34 |
| GP-3 | 84.00 | 81.44 | 56.00 | 50.98 | 24.00 | 50.00 | 44.00 | 40.00 | 50.00 | 31.03 | 28.00 | 16.67 | 47.67 | 45.02 |
| GP-4 | 64.00 | 47.90 | 66.00 | 62.75 | 20.00 | 35.00 | 40.00 | 20.00 | 44.00 | 41.38 | 74.00 | 55.56 | 51.33 | 43.76 |
| VGG19 | 98.00 | 89.22 | 90.00 | 86.27 | 76.00 | 55.00 | 56.00 | 80.00 | 72.00 | 82.76 | 62.00 | 61.11 | 75.67 | 75.73 |
| ResNet101 | 100.00 | 85.03 | 94.00 | 84.31 | 78.00 | 65.00 | 62.00 | 53.33 | 76.00 | 72.41 | 68.00 | 66.67 | 79.67 | 71.13 |
| ResNet152 | 98.00 | 92.22 | 92.00 | 86.27 | 72.00 | 65.00 | 74.00 | 80.00 | 72.00 | 65.52 | 62.00 | 66.67 | 78.33 | 75.95 |
| SeNet154 | 98.00 | 92.22 | 92.00 | 86.27 | 68.00 | 45.00 | 66.00 | 66.67 | 80.00 | 75.86 | 62.00 | 72.22 | 77.67 | 73.04 |
| DPN92 | 98.00 | 91.02 | 92.00 | 86.27 | 80.00 | 40.00 | 64.00 | 73.33 | 78.00 | 75.86 | 66.00 | 61.11 | 79.67 | 71.27 |
| Xception | 100.00 | 83.83 | 92.00 | 82.35 | 72.00 | 65.00 | 64.00 | 73.33 | 78.00 | 79.31 | 56.00 | 66.67 | 77.00 | 75.08 |
| InceptionV3 | 100.00 | 88.62 | 92.00 | 88.24 | 68.00 | 60.00 | 60.00 | 86.67 | 78.00 | 72.41 | 60.00 | 38.89 | 76.33 | 72.47 |
| InceptionResnetV2 | 98.00 | 86.83 | 88.00 | 90.20 | 72.00 | 60.00 | 60.00 | 66.67 | 72.00 | 65.52 | 64.00 | 55.56 | 75.67 | 70.79 |
| NASNetAMobile | 96.00 | 77.84 | 92.00 | 78.43 | 70.00 | 55.00 | 68.00 | 53.33 | 64.00 | 65.52 | 60.00 | 66.67 | 75.00 | 66.13 |
| PNASNetLarge | 100.00 | 91.62 | 90.00 | 84.31 | 70.00 | 60.00 | 54.00 | 73.33 | 74.00 | 79.31 | 60.00 | 66.67 | 74.67 | 75.87 |
| InceptionV4 | 100.00 | 87.43 | 96.00 | 86.27 | 80.00 | 60.00 | 70.00 | 80.00 | 76.00 | 82.76 | 56.00 | 55.56 | 79.67 | 75.34 |
| Densenet201 | 100.00 | 89.22 | 90.00 | 86.27 | 78.00 | 50.00 | 62.00 | 73.33 | 66.00 | 79.31 | 62.00 | 61.11 | 76.33 | 73.21 |
| Ensemble† | 100.0 | 92.81 | 94.00 | 86.27 | 76.00 | 60.00 | 76.00 | 80.00 | 74.00 | 79.32 | 64.00 | 77.78 | 80.33 | 79.36 |

Table S5. Comparison of human and machine learning model’s F1-score. Overall F1 score is macro-average F1 score.†Ensemble: Ensemble of ResNet152, DPN92, InceptionV4, Densenet201 model. Results not included to CNN statistics. T_Bal_: Balanced test set, T_Imbal_: Imbalanced test set. ENT: otolaryngologists; ENT-R: otolaryngology residents (numbers indicate years of training); FM: family medicine specialists; EM: emergency medicine specialists; GP: general practitioners. CNN: convolutional neural network. Other abbreviations are same as Figure 1

| Reviewer | Per-class F1 score | | | | | | | | | | | | Overall  F1-score | |
| --- | --- | --- | --- | --- | --- | --- | --- | --- | --- | --- | --- | --- | --- | --- |
|  | No | | Tp | | Ar | | Oe | | Om | | Tu | |  |  |
|  | T_Bal_ | T_Imbal_ | T_Bal_ | T_Imbal_ | T_Bal_ | T_Imbal_ | T_Bal_ | T_Imbal_ | T_Bal_ | T_Imbal_ | T_Bal_ | T_Imbal_ | T_Bal_ | T_Imbal_ |
| ENT-1 | 66.67 | 90.53 | 92.78 | 92.16 | 66.67 | 62.50 | 73.33 | 50.00 | 73.87 | 66.67 | 77.55 | 76.47 | 75.15 | 73.05 |
| ENT-2 | 70.27 | 83.13 | 87.62 | 90.91 | 62.79 | 37.04 | 55.00 | 26.09 | 68.42 | 51.28 | 69.23 | 73.33 | 68.89 | 60.30 |
| ENT-R4 | 73.68 | 79.07 | 90.91 | 86.32 | 62.22 | 69.77 | 65.22 | 43.48 | 67.47 | 40.48 | 67.96 | 64.52 | 71.24 | 63.94 |
| ENT-R3 | 76.19 | 83.12 | 89.36 | 86.60 | 68.09 | 57.14 | 70.27 | 35.71 | 71.56 | 44.44 | 75.86 | 75.00 | 75.22 | 63.67 |
| ENT-R2 | 65.73 | 80.13 | 81.82 | 78.72 | 78.72 | 59.46 | 60.38 | 25.97 | 75.27 | 40.00 | 52.63 | 60.00 | 69.09 | 57.38 |
| ENT-R1 | 59.68 | 81.01 | 84.21 | 68.35 | 57.83 | 50.00 | 61.67 | 25.53 | 63.64 | 51.76 | 75.56 | 55.17 | 67.10 | 55.31 |
| FM-1 | 71.43 | 81.03 | 72.73 | 82.98 | 37.97 | 40.00 | 43.14 | 26.32 | 61.40 | 30.77 | 57.14 | 50.00 | 57.30 | 51.85 |
| FM-2 | 52.94 | 59.29 | 80.85 | 59.34 | 40.96 | 15.87 | 41.67 | 19.51 | 32.94 | 30.43 | 46.55 | 24.62 | 49.32 | 34.84 |
| EM-1 | 49.59 | 31.68 | 65.17 | 65.12 | 41.74 | 18.67 | 5.97 | 9.09 | 20.00 | 24.00 | 60.94 | 26.26 | 40.57 | 29.14 |
| EM-2 | 51.92 | 68.68 | 35.48 | 42.67 | 33.61 | 28.57 | 45.51 | 18.18 | 56.18 | 46.15 | 20.34 | 28.57 | 40.51 | 38.80 |
| GP-1 | 23.08 | 17.11 | 52.27 | 59.09 | 32.52 | 10.99 | 24.78 | 10.53 | 32.08 | 28.24 | 26.09 | 5.48 | 31.80 | 21.91 |
| GP-2 | 44.90 | 36.02 | 66.67 | 59.34 | 45.36 | 17.86 | 20.45 | 13.46 | 20.87 | 23.88 | 46.60 | 30.99 | 40.81 | 30.26 |
| GP-3 | 57.93 | 80.00 | 65.88 | 56.52 | 23.53 | 31.25 | 40.37 | 41.38 | 54.95 | 37.50 | 41.18 | 22.22 | 47.31 | 44.81 |
| GP-4 | 49.23 | 62.50 | 66.00 | 64.00 | 29.85 | 16.67 | 43.96 | 16.67 | 46.81 | 35.82 | 62.71 | 35.09 | 49.76 | 38.46 |
| VGG19 | 79.67 | 91.98 | 83.33 | 83.02 | 77.55 | 53.66 | 65.88 | 80.00 | 72.00 | 76.19 | 72.09 | 61.11 | 75.09 | 74.33 |
| ResNet101 | 79.37 | 88.75 | 87.04 | 76.11 | 82.11 | 66.67 | 72.94 | 55.17 | 80.85 | 68.85 | 73.91 | 63.16 | 79.37 | 69.78 |
| ResNet152 | 77.17 | 92.49 | 86.79 | 85.44 | 79.12 | 66.67 | 78.72 | 70.59 | 73.47 | 69.09 | 73.81 | 66.67 | 78.18 | 75.16 |
| SeNet154 | 79.03 | 91.67 | 85.98 | 80.73 | 70.10 | 54.55 | 75.86 | 64.52 | 81.63 | 78.57 | 71.26 | 74.29 | 77.31 | 74.05 |
| DPN92 | 82.35 | 92.68 | 85.98 | 80.00 | 80.00 | 53.33 | 73.56 | 64.71 | 79.59 | 70.97 | 74.16 | 61.11 | 79.27 | 70.47 |
| Xception | 84.03 | 88.61 | 85.98 | 72.41 | 73.47 | 63.41 | 70.33 | 73.33 | 76.47 | 79.31 | 67.47 | 61.54 | 76.29 | 73.10 |
| InceptionV3 | 80.65 | 90.52 | 85.98 | 80.36 | 73.91 | 64.86 | 68.18 | 70.27 | 78.00 | 71.19 | 67.42 | 50.00 | 75.69 | 71.20 |
| InceptionResnetV2 | 78.40 | 89.51 | 84.62 | 77.97 | 73.47 | 64.86 | 68.18 | 57.14 | 74.23 | 70.37 | 72.73 | 62.50 | 75.27 | 70.39 |
| NASNetAMobile | 81.36 | 84.14 | 85.98 | 69.57 | 72.92 | 48.89 | 70.10 | 43.24 | 68.09 | 69.09 | 68.18 | 61.54 | 74.44 | 62.74 |
| PNASNetLarge | 76.34 | 91.62 | 80.36 | 83.50 | 76.92 | 64.86 | 60.67 | 66.67 | 79.57 | 80.70 | 71.43 | 66.67 | 74.21 | 75.67 |
| InceptionV4 | 84.03 | 91.25 | 86.49 | 82.24 | 80.81 | 60.00 | 69.31 | 68.57 | 84.44 | 77.42 | 70.00 | 55.56 | 79.18 | 72.51 |
| Densenet201 | 78.13 | 91.13 | 78.26 | 80.73 | 82.11 | 57.14 | 72.09 | 64.71 | 74.16 | 79.31 | 71.26 | 59.46 | 76.00 | 72.08 |
| Ensemble† | 84.03 | 93.37 | 83.64 | 83.02 | 82.11 | 66.67 | 74.47 | 77.42 | 80.00 | 80.70 | 75.86 | 73.68 | 80.02 | 79.14 |
